# Supplementary material for: Client-centered counseling and facilitation in improving modern contraceptive uptake in urban slum of Karachi Pakistan
Source: PLoS One. 2023 Jul 31;18(7):e0289107. doi: 10.1371/journal.pone.0289107 (PMC10389712; doi:10.1371/journal.pone.0289107)
Supplement: Attachment — Submitted filename: Point by point response.docx [file pone.0289107.s001.docx]

**Point by point response**

**PONE-D-22-17428**

Reproductive Health for Women in Need – A Novel Perspective of a Case from Urban Slum of Karachi Using Client-centered Counseling and Facilitation of High-risk Women in Improving Modern Contraceptive Prevalence Rate

| **S. No.** | **Comments** | **Response** |
| --- | --- | --- |
| **Journal Requirements** | | |
| 1. | Please ensure that your manuscript meets PLOS ONE's style requirements, including those for file naming. The PLOS ONE style templates can be found at | Thanks. We have formatted the manuscript according to the guidelines now. |
| 2. | Thank you for stating the following financial disclosure:   “No funding”  At this time, please address the following queries:  a)        Please clarify the sources of funding (financial or material support) for your study. List the grants or organizations that supported your study, including funding received from your institution.  b)        State what role the funders took in the study. If the funders had no role in your study, please state: “The funders had no role in study design, data collection and analysis, decision to publish, or preparation of the manuscript.”  c)        If any authors received a salary from any of your funders, please state which authors and which funders.  d)        If you did not receive any funding for this study, please state: “The authors received no specific funding for this work.”  Please include your amended statements within your cover letter; we will change the online submission form on your behalf. | We have updated the information in the Declaration section, page 9, under Funding |
| 3. | Thank you for stating the following in your Competing Interests section:  “No author have competing interests”  Please complete your Competing Interests on the online submission form to state any Competing Interests. If you have no competing interests, please state "The authors have declared that no competing interests exist.", as detailed online in our guide for authors at <http://journals.plos.org/plosone/s/submit-now>   This information should be included in your cover letter; we will change the online submission form on your behalf. |  |
| 4. | Please amend the manuscript submission data (via Edit Submission) to include authors Rashid Ali and Alishan Bachlany. | We have corrected the authors information appropriately. There was error, which was done on our behalf. |
| 5. | Your ethics statement should only appear in the Methods section of your manuscript. If your ethics statement is written in any section besides the Methods, please move it to the Methods section and delete it from any other section. Please ensure that your ethics statement is included in your manuscript, as the ethics statement entered into the online submission form will not be published alongside your manuscript | Done. |
| 6. | Please include your tables as part of your main manuscript and remove the individual files. Please note that supplementary tables (should remain/ be uploaded) as separate "supporting information" files" | Done |
| **Reviewers' comments** | | |
| 7. | Figure 1 is not clearly visible and should be improved. | Apologies for this, we have now made it clearer. |
| 8. | The maternal education is a binary variable with two categories "no education" and "at least primary". These categories are not exhaustive and do not include mothers with 1-5 education levels. This variable needs to be re- constructed. | Thanks, we agree with the reviewer point. However, we created this binary variable since very few MWRA among this most vulnerable cohort had education level of secondary (n=277) and none have above secondary. So based one some other research work from the similar context, we created this binary variable. We have now changed the term to “Have some formal education” instead of ‘at least primary’. Page 7 and table 2. Further, we provided this detail in Table 1 as descriptive. |
| 9. | Conclusion section needs to be improved. The objectives of the study should be matched with its findings and mention limitations, if any. | Many thanks. We agree and try to improve it further. Please check page 9 |
| 10. | Objectives of the study are vague and not stated clearly. | We are thankful that you raised this point. To improve it and make it clearer we have restructured it. Page 4, line 89-103 |
| 11. | Methodology is not clear | Thanks. We have added more detail, please check section Methods, page 5, line 115-135 |
| 12. | Results of the study do not match the mentioned objectives | Thanks, as we have improved the objectives, we are hoping that now they are consistent with the redefined/restructured objectives. |
