# Supplementary material for: Client-centered counseling and facilitation in improving modern contraceptive uptake in urban slum of Karachi Pakistan
Source: PLoS One. 2023 Jul 31;18(7):e0289107. doi: 10.1371/journal.pone.0289107 (PMC10389712; doi:10.1371/journal.pone.0289107)
Supplement: Attachment — Submitted filename: Point by point response-12May2023.docx [file pone.0289107.s002.docx]

**Point by point response**

**PONE-D-22-17428**

Client-centered Counseling and Facilitation in Improving Modern Contraceptive Uptake in Urban slum of Karachi Pakistan

| **S. No.** | **Comments** | **Response** |
| --- | --- | --- |
| **Journal Requirements** | | |
| 1. | We strongly propose a major revision of this article as there are major flaws. the objectives are not addressed in results and are not clear. | We apologize for the confusion and for not clarifying it in the beginning. We have improved the section and aligned it with the analysis. The main confusion was related to the term the term “targeted” which in true sense was related to “promotion of LARC” promotion of LARC and framed for well the previous version. We have now reworded the objectives as: “The aims of this paper are: (i) to present the adoption rates of overall modern contraceptive methods as well as LARC or permanent methods after the implementation of client-centered counseling and facilitation by FP service providers; and (ii) to delineate the sociodemographic and maternal factors and the reasons that account for the insufficient adoption rates despite the provision of counseling services”. Page 5, line 93. Further, we have improved the sections in the introduction, methods, results, and discussion to make it more coherent for the reviewers and audience of this paper. These changes can be observed in the tracked changes. |
