# Supplementary material for: Client-centered counseling and facilitation in improving modern contraceptive uptake in urban slum of Karachi Pakistan
Source: PLoS One. 2023 Jul 31;18(7):e0289107. doi: 10.1371/journal.pone.0289107 (PMC10389712; doi:10.1371/journal.pone.0289107)
Supplement: Attachment — Submitted filename: Point by point response.docx [file pone.0289107.s003.docx]

**Point by point response**

**PONE-D-22-17428**

Client-centered Counseling and Facilitation in Improving Modern Contraceptive Uptake in Urban slum of Karachi Pakistan

| **S. No.** | **Comments** | **Response** |
| --- | --- | --- |
| **Journal Requirements** | | |
| 1. | The title on cover letter does not match with original article title. kindly correct it | Apologies, we have corrected it  . |
| 2. | Please explain the client centered approach in detail. just listening and involving women in decision making does not work in our community where men have empowerment. what innovation are you bringing through this paper in previous literature | Thanks. We have added more information about the approach in the methodology section, in the yellow highlight text. Page 5 and 6. We have also provided information with regards to the involvement of various key decision players at household level.  We also explained that compared to other programs/projects which were implemented in the same or adjacent setting, our findings are unique. Please note that our approach has aim to increase the uptake of LARC, which we were successfully able to achieve. Please see information in discussion section |
| 3. | Most of the findings are the same as already present in literature. kindly highlight your innovation | We have added a few more information in the discussion section, the key finding in our case is increase uptake of implant and IUCD, which was missing in previous studies from same setting. Page 10 and 12 |
| 4. | What new message are you giving to your reader? | We have added few more information in discussion (page 12) and conclusion |
| 5. | Way forward? | We have made changes in the conclusion |
